# Association between tooth loss and handgrip strength in a general adult population

**DOI:** 10.1371/journal.pone.0236010

**Published:** 2020-07-10

**Authors:** Ziqi Zhou, Yeqing Gu, Qing Zhang, Li Liu, Hongmei Wu, Ge Meng, Xue Bao, Shunming Zhang, Shaomei Sun, Xing Wang, Ming Zhou, Qiyu Jia, Kun Song, Yue Zhao, Kaijun Niu

**Affiliations:** 1 School of Nursing, Tianjin Medical University, Tianjin, China; 2 Nutritional Epidemiology Institute and School of Public Health, Tianjin Medical University, Tianjin, China; 3 Health Management Centre, Tianjin Medical University General Hospital, Tianjin, China; 4 Tianjin Key Laboratory of Environment, Nutrition and Public Health, Tianjin, China; 5 Center for International Collaborative Research on Environment, Nutrition and Public Health, Tianjin, China; University of Tennessee Health Science Center College of Graduate Health Sciences, UNITED STATES

## Abstract

Tooth loss is a prevailing condition in China due to the high prevalence of oral diseases. Since previous studies explored the association between tooth loss and handgrip strength showed incongruous results, the aim of this study was to investigate the association between tooth loss and handgrip strength in Tianjin, China. Cross-sectional data in the present study used baseline data of Tianjin Chronic Low-grade Systemic Inflammation and Health (TCLSIH) Cohort Study during 2013–2016. Eligible adults (n = 26275) were classified into four groups depending on the number of missing teeth (excluding third molars): 0, 1–2, 3–5 and ≥6. Handgrip strength was measured using a handheld type dynamometer. Analysis of covariance was used to examine the relationships between tooth loss and handgrip strength and handgrip strength per body weight according to gender. After adjustment for potential confounders, the relationships existed between increasing categories of tooth loss and handgrip strength, as well as handgrip strength per weight. The data of stratified analysis showed that there was a trending association between decreased handgrip strength and fewer retained teeth both in males and females less than 60 years of age (*P* for trend <0.01); whereas no significant association 60 years of age or older. Moreover, loss of more than 3 teeth was significantly associated with reduced muscle strength (*P* <0.01). Tooth loss is independently associated with handgrip strength in Chinese adults less than 60 years of age.

## Introduction

Tooth loss is a prevailing condition all over the world contributed by a number of factors, among which periodontitis and caries are the main causes [1, 2]. Recent data from The Fourth National Epidemiology Survey of Oral Health reported that 96.7% of Chinese adults aged between 35–44 have dental calculus, 87.4% have gingival bleeding [3], both higher compared to 10 years ago [3, 4]. This indicates that Chinese adults are still in poor oral state, and tooth loss as the denouement is still problematic. Handgrip strength which is an accurate and easily assessed estimating measure of muscle strength, is an important indicator for physical performance and nutritional status of adults [5, 6]. Reduced handgrip strength is associated with increased disability [7–9] and mortality [10, 11]. In addition, handgrip strength is a powerful predictor of sarcopenia [12] and frailty [13] among elderly. In this study, handgrip strength has been evaluated as an estimate of overall muscle strength [5].

The association between tooth loss and several chronic diseases or conditions such as obesity [14–16], cardiovascular diseases [14, 17], diabetes [18, 19] and metabolic syndrome [20] has been well documented. A possible mechanism by which tooth loss is associated with systemic diseases is the inflammatory pathway. Tooth loss is resulting from previous or current oral bacterial infection such as periodontal (gingival) diseases and carious lesions [21]. Thus, the number of missing teeth may reflect cumulative level of oral inflammation [22], a common source of low-grade systemic inflammation leads to increasing levels of inflammatory cytokines [23, 24], probably being a link to chronic diseases [25]. On the other hand, studies have suggested that elevated levels of inflammatory cytokine brought by chronic low-grade inflammation were associated with loss of strength [26]. Inflammatory cytokines also affect the synthesis and secretion of anabolic hormones [such as testosterone and insulin-like growth factor 1 (IGF-1)] on both muscle mass and strength [27, 28]. Moreover, tooth loss in adults may affect muscle status through lower dietary quality and reduced intake of most nutrients [1, 29]. Therefore, the relationship between tooth loss and handgrip strength can be hypothesized.

A few researchers have studied the association between tooth loss and muscle strength, most of them have only focused on adults in old age [30–33]. Moreover two studies failed to find an association [33, 34] and one study only find the association in men [30]. Therefore, studies in a general adult population to determine whether tooth loss is associated with handgrip strength are required to support the previous findings and expand the generalizability.

The aim of this study is to investigate the relationship between the number of missing teeth and muscle strength represented by handgrip strength in a general adult population. The data we used was the baseline data from the Tianjin Chronic Low-grade Systemic Inflammation and Health (TCLSIH or TCLSIHealth) Cohort Study.

## Materials and methods

### Study design and participants

This cross-sectional study used data from TCLSIH Cohort Study, a prospective dynamic cohort study designed to investigate the relationship between chronic low-grade systemic inflammation and health status of the general population in Tianjin, China [35]. TCLSIH sample was recruited from several hospital health management centers and community management centers, and the participants underwent their annual health examination which generally included items of anthropometric parameters [weight, height, waist circumference (WC)], blood pressure (BP), blood biochemical examination [fasting blood glucose (FBG), triglycerides (TG), total cholesterol (TC), low-density lipoprotein cholesterol (LDL-C) and high-density lipoprotein cholesterol (HDL-C)] and physical performance (handgrip strength) there. Meanwhile, randomly selected participants had completed a standardized structured interview questionnaire which including but not limited to age, sex, employment status, educational level, marital status, family income, dental condition, physical activity (PA), dietary habits, diseases and use of medicines since May 2013. The TCLSIH Cohort Study was approved by Tianjin Medical University Institutional Review Board and written informed consent was obtained from all participants.

The baseline data of the TCLSIH Cohort Study (2013–2016) was used in the present study. Participants who completed the health examination and questionnaire were initially included (n = 28131). Among them, 1856 participants excluded as they met the exclusion criteria: were under the age of 18 (n = 21); had missing information in handgrip strength and dental condition part (n = 158); had a history of cardiovascular disease (n = 1396) [10, 36, 37] or cancer (n = 281) [38, 39], because of these conditions affected muscle strength. The final study population comprised 26275 participants.

### Assessment of missing teeth

The number of missing teeth was obtained from the question in the questionnaire, there were four alternative categories depending on it: 0 (full dentition, 28 teeth), 1–2 (26–27 teeth), 3–5 (23–25 teeth) and ≥6 (22 teeth or less), participants selected and recorded. In the present study, 3 missing teeth was used as a cut-off value. According to the data of National Health and Nutrition Examination Survey, the average number of missing teeth between the ages of 35 and 49 is 3 (NIDCR, 2018). And the average age of the present study participants was 41.6 years.

### Assessment of handgrip strength

Generally, handgrip strength is measured to estimate muscle strength. Participants were tested under the same conditions by trained technicians. We used a handheld type dynamometer (EH101; CAMRY) which was calibrated before each survey, error range within 0.1 kg. Before the measurement, adjusted the dynamometer width for each participant’ palm. The maximal voluntary handgrip strength of each hand was measured in an upright position and the better score was recorded as the final handgrip strengths, which was indicated in kg. Handgrip strength relative to body weight (kg/kg) was also calculated because handgrip strength is affected by body weight [40].

### Assessment of other variables

Weight and height were measured by a height and weight meter and WC by a tape using the standard method. Body mass index (BMI) was calculated as weight /height squared. BP was measured from the upper left arm using an automatic digital sphygmomanometer (KD598; Andon) in a seated position. Blood samples were drawn in fasting state from veins and all blood biochemical tests were performed using a Cobas 8000 analyzer with Roche original reagents. The diagnostic criteria of metabolic syndrome (MS) were using the criteria of the American Heart Association scientific statements of 2009 [41].

Physical activity (PA) was assessed using the short version of International Physical Activity Questionnaire. Activities were divided into three activity levels: walking; moderate level of activity (such as child care or household activity) and vigorous level of activity (such as swimming, running, or other sports activities). Participants answered the questionnaire based on the duration of activities they had performed during the most recent week. Metabolic equivalent (MET) hours were calculated using corresponding MET coefficients (3.3 for walking, 4.0 for moderate level of activity and 8.0 for vigorous level of activity) according to the following formula: MET coefficients × duration (hours) × frequency (days) [42]. Total PA was the sum of the above three physical activities’ scores.

A semi-quantitative Food Frequency Questionnaire was used to evaluate the dietary patterns of participants which contains 81 items of food and beverages [43]. The food consumption frequency includes 7 options: 2 times a day, 1 time a day, 4–6 times a week, 2–3 times a week, 1 time a week, < 1 time a week, almost never; the beverage consumption frequency includes 8 options: 2–4 cups per day, 2- cups per day, 1 cup per day, 4–6 cups per week, 2–3 cups per week, 1 cup per week, < 1 cup per week, almost never. The participants were required to make a choice based on their consumption frequency within a month. The average daily consumption of each item was calculated by an ad hoc computer program developed to analyze the questionnaire. Factor analysis was used and three dietary patterns which named ‘‘fruits and sweets” pattern, ‘‘health” pattern, and ‘‘animal foods” pattern were derived.

Other socio-demographic data were also obtained from the same questionnaire. The educational level was sorted into two categories: < college graduate or ≥ college graduate; the family income was sorted into two categories: < or > 10000 Yuan. Employment status was defined as managers, professionals and others. Smoking status was defined as ‘smoker’, ‘ex-smoker’, and ‘non-smoker’ and drinking status was defined as ‘everyday’, ‘occasional’, and ‘ex-drinker’ and ‘non-drinker’.

### Statistical analysis

All the statistical analyses were performed by gender, as the difference in muscle strength between men and women was significant (*P*<0.001). Kolmogorov-Smirnov (n>2000) or Shapiro-Wilk (n≤2000) were used to test the normality of continuous variables. The results showed that the distribution of most continuous variables in this study was not normal. Therefore, before statistical analyses, natural logarithm was used to normalize continuous variables, and after logarithm transformation the continuous variables were approached normal distribution. Adjusted continuous variables were expressed by geometric mean [95% confidence interval (CI)], and classified variables were expressed by percentage. In the baseline characteristics analyses, analysis of variance (ANOVA) were performed for continuous variables and logistic regression analysis were performed for classified variables to test the differences across tooth loss categories (according to the number of missing teeth, 0:1; 1–2:2, 3–5:3, ≥6:4). For analysis, the handgrip strength was used as dependent variable, and tooth loss categories were used as independent variable. Bonferroni-corrected *P* values were used to compare the differences of handgrip strength between tooth loss categories. Variance inflation factors (VIFs) were used for multicollinearity test, and VIFs less than 10 indicated that there was no collinearity. Relationship between tooth loss categories and handgrip strength were examined using analysis of covariance (ANCOVA) by three different models. Model 1 was crude, model 2 was adjusted for age, WC and BMI, and model 3 was additionally adjusted for family income, educational levels, employment status, smoking status, drinking status, PA, total energy intake, dietary patterns, MS. Interactions between tooth loss categories and age were tested by the addition of cross-product terms in males and females, respectively, and both interaction terms were significant (*P* <0.0001 both in males and females). Thus, we performed a stratified analysis, stratifying on age less than 60 years of age versus age greater than or equal to 60 years [44]. All statistical tests were performed by bilateral test, and *P*<0.05 was defined as statistically significant. All analyses were performed using the Statistical Analysis System 9.3 edition for Windows (SAS Institute Inc.).

## Results

### Characteristics of participants

26275 participants were included in this study with age ranging between 18–90 years, mean ages (standard deviation, SD) was 41.6 (11.9) years, among which 13983 (53.2%) were males and 12292 (46.8%) were females. Excluding third molars, 16266 (61.91%) participants had full dentition, 5961 (22.69%) had 26–27natural teeth, 2829 (10.77%) had 23–25natural teeth, and 1219 (4.64%) had 22 natural teeth or less. The mean handgrip strength (SD) was 43.06 (1.18) kg in male, and 25.43 (1.20) kg in female. There were significant differences (P<0.001), so all analyses were performed according to gender.

Table 1 summarizes participants’ characteristics according to their tooth loss categories. The analysis were showed as ANOVA for continuous variables and logistic regression analysis for categorical variables. Compared with participants having full dentition, participants with increasingly missing teeth tended to be older and to have higher PA and WC. Among the participants with more missing teeth, there were a higher proportion of smokers, alcohol consumers, MS patients, and a lower proportion of college graduate, managers, professionals in both men and women (*P* for trends all<0.0001). In addition, there was no significant difference between tooth loss and energy intake.

**Table 1:** Participant characteristics by tooth loss status^a^.

Number of missing teeth					
	0	1–2	3–5	≥6	*P* for trend^b^
**Males(n = 13983)**	8,400	3,265	1,592	726	-
Age (y)	36.8 (36.6, 37.0)^c^	43.9 (43.6, 44.3)	50.5 (49.9, 51.1)	58.1 (57.1, 59.1)	<0.0001
BMI (kg/m^2^)	25.6 (25.5, 25.7)	25.6 (25.5, 25.8)	25.7 (25.6, 25.9)	25.4 (25.2, 25.7)	0.089
WC (cm)	87.8 (87.6, 88.0)	88.7 (88.4, 89.0)	89.5 (89.0, 90.0)	89.4 (88.7, 90.1)	<0.0001
MS (%)	31.6	39.6	43.2	45.6	<0.0001
PA (Mets × hour/week)	11.3 (11.0, 11.6)	11.4 (10.9, 12.0)	12.7 (11.9, 13.6)	15.8 (14.3, 17.4)	<0.001
Energy intake (kcal/d)	2074.2 (2062.0, 2086.4)	2070.1 (2050.6, 2089.7)	2065.8 (2038.0, 2093.9)	2082.7 (2041.1, 2124.9)	0.59
“Fruits and sweets” dietary pattern (factor score)	0.78 (0.76, 0.79)	0.78 (0.76, 0.81)	0.75 (0.72, 0.78)	0.77 (0.73, 0.82)	0.089
“Healthy” dietary pattern (factor score)	0.86 (0.84, 0.88)	0.89 (0.86, 0.93)	0.97 (0.93, 1.02)	1.10 (1.02, 1.18)	<0.0001
“Animal foods” dietary pattern (factor score)	0.95 (0.94, 0.97)	0.91 (0.89, 0.94)	0.85 (0.82, 0.89)	0.76 (0.71, 0.80)	<0.0001
Smoking status (%)					
Smoker	34.6	39.7	45.0	42.9	<0.0001
Ex-smoker	7.71	10.2	12.9	17.4	<0.0001
Non-smoker	57.7	50.1	42.2	39.8	<0.0001
Drinking status (%)					
Everyday	6.61	9.31	14.7	19.0	<0.0001
Occasional	73.6	70.2	63.9	54.7	<0.0001
Ex-drinker	9.67	9.86	11.1	10.5	0.12
Non-drinker	10.2	10.6	10.4	15.8	<0.001
Educational level (≥college graduate, %)	75.1	64.0	48.5	36.0	<0.0001
Employment status (%)					
Managers	45.9	46.5	43.3	35.1	<0.0001
Professionals	19.7	18.7	17.1	12.3	<0.0001
Others	34.4	34.8	39.6	52.6	<0.0001
Family income (≥10000 Yuan, %)	36.7	34.1	27.5	20.8	<0.0001
**Females(n = 12292)**	7,866	2,696	1,237	493	-
Age (y)	36.0 (35.8, 36.2)	41.9 (41.5, 42.3)	47.4 (46.8, 48.1)	56.7 (55.5, 58.0)	<0.0001
BMI (kg/m^2^)	22.4 (22.4, 22.5)	23.2 (23.1, 23.3)	23.5 (23.3, 23.7)	24.7 (24.4, 25.1)	<0.0001
WC (cm)	74.3 (74.1, 74.5)	76.6 (76.3, 76.9)	78.2 (77.7, 78.8)	82.8 (82.0, 83.7)	<0.0001
MS (%)	10.8	18.1	23.7	38.3	<0.0001
PA (Mets × hour/week)	8.68 (8.44, 8.90)	9.28 (8.83, 9.80)	10.3 (9.57, 11.1)	13.4 (11.9, 15.0)	<0.0001
Energy intake (kcal/d)	1919.7 (1906.7, 1932.9)	1936.6 (1914.1, 1959.3)	1940.6 (1907.5, 1974.4)	1941.1 (1888.8, 1994.8)	0.25
“Fruits and sweets” dietary pattern (factor score)	0.84 (0.83, 0.86)	0.88 (0.85, 0.90)	0.89 (0.86, 0.92)	0.92 (0.87, 0.98)	<0.01
“Healthy” dietary pattern (factor score)	0.69 (0.68, 0.71)	0.74 (0.71, 0.77)	0.76 (0.72, 0.81)	0.91 (0.83, 1.00)	<0.01
“Animal foods” dietary pattern (factor score)	0.65 (0.64, 0.66)	0.60 (0.58, 0.62)	0.58 (0.56, 0.61)	0.46 (0.42, 0.49)	<0.001
Smoking status (%)					
Smoker	0.94	1.43	1.79	4.49	<0.0001
Ex-smoker	0.68	0.51	0.72	1.57	0.24
Non-smoker	98.4	98.0	97.5	93.9	<0.0001
Drinking status (%)					
Everyday	0.56	0.94	1.07	1.45	<0.01
Occasional	39.4	40.7	39.1	31.9	0.08
Ex-drinker	10.4	9.70	7.88	7.66	<0.01
Non-drinker	49.6	48.7	51.9	59.0	<0.01
Educational level (≥college graduate, %)	73.2	59.0	46.7	27.0	<0.0001
Employment status (%)					
Managers	44.3	41.0	38.1	21.8	<0.0001
Professionals	12.7	12.2	11.6	8.89	0.02
Others	43.0	46.7	50.4	69.3	<0.0001
Family income (≥10000 Yuan, %)	35.1	32.1	25.9	17.6	<0.0001

^a^ BMI, body mass index; WC, waist circumference

^b^ Analysis of variance or logistic regression analysis.

^c^ Geometric least square mean (95% confidence interval) (all such values).

### Association between tooth loss and handgrip strength

**Fig 1:**
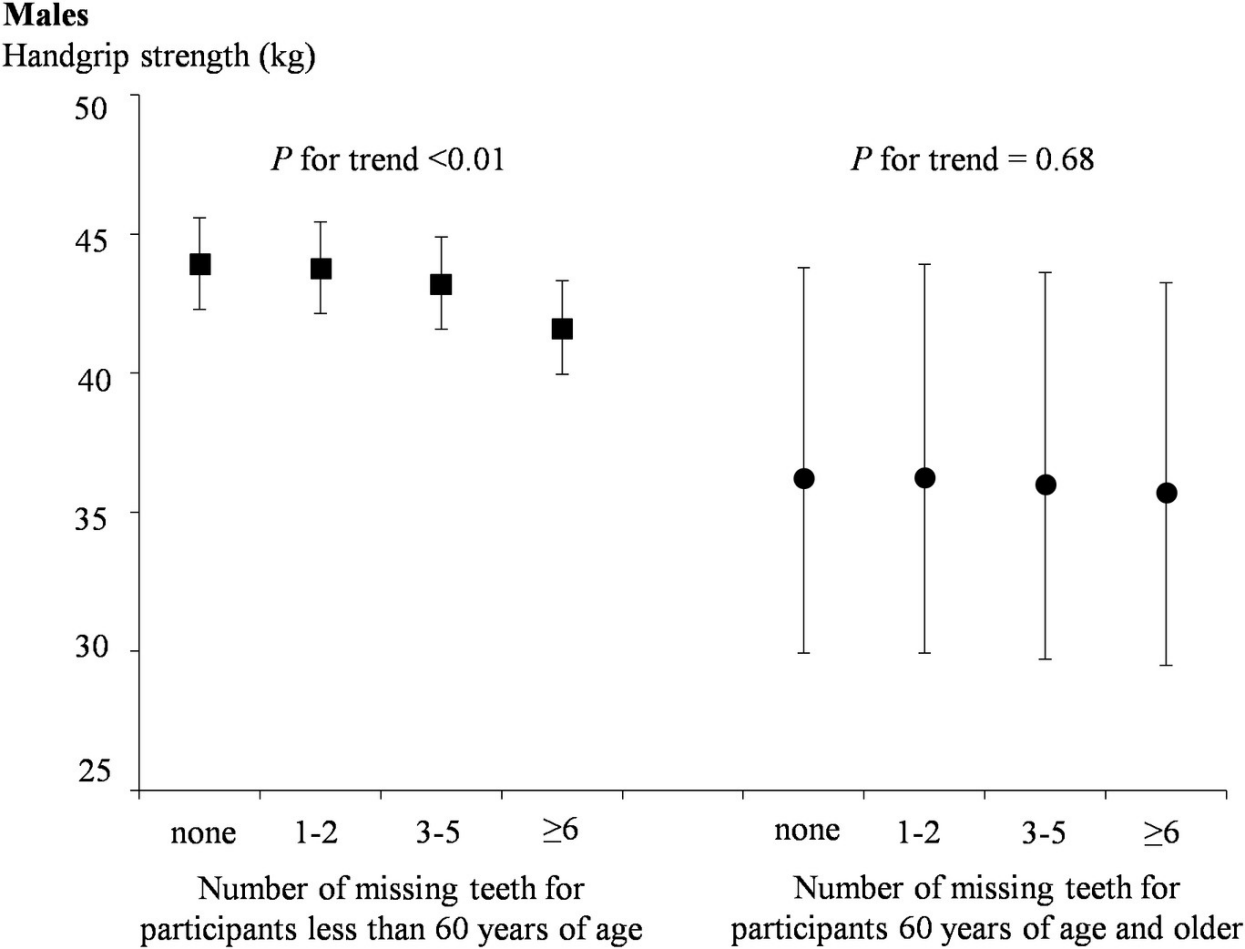
Adjusted relationships of tooth loss status with handgrip strength by age for males.

**Fig 2:**
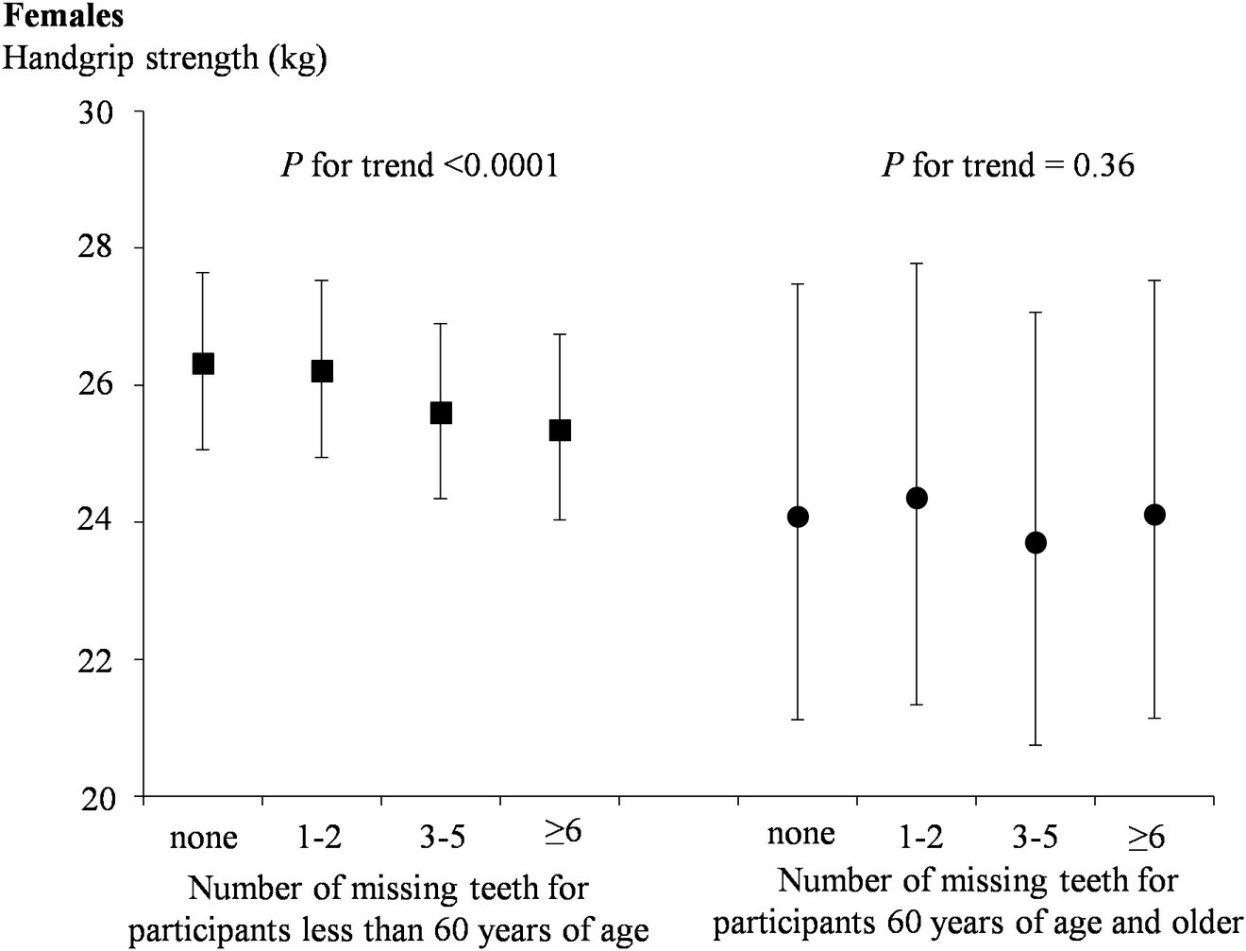
Adjusted relationships of tooth loss status with handgrip strength by age for females.

Table 2 shows the gender-specific relationship between tooth loss categories and handgrip strength, as well as handgrip strength per weight of three models. Model 1 indicated an association between tooth loss and handgrip strength, as well as handgrip strength per weight (*P* for trend < 0.0001). In model 2 and model 3, after adjusting for several variables, the association remained statistically significant in both males and females (*P* for trend < 0.0001). The adjusted means [95% confidence interval (CI)] for handgrip strength (kg) in males and females across increasing categories of tooth loss were 43.67(42.15, 45.25), 43.63(42.11, 45.22), 42.76(41.24, 44.34) and 40.26(38.79, 41.79); 26.00(24.84, 27.21), 25.95(24.79, 27.17), 25.17(24.03, 26.37) and 24.38(23.24, 25.58) (all *P* for trend <0.0001), respectively. The adjusted means (95% CI) for handgrip strength per weight (kg/kg) in males and females across increasing categories of tooth loss were 0.57(0.55, 0.59), 0.57(0.55, 0.59), 0.56(0.54, 0.58), and 0.53(0.51, 0.55); 0.43(0.41, 0.45), 0.43(0.41, 0.45), 0.42(0.40, 0.43), and 0.41(0.39, 0.43) (all *P* for trend <0.0001), respectively. We also investigated the difference of handgrip strength among different categories of tooth loss as shown in Table 2. Handgrip strength was significantly different between the group with 0 missing teeth, the group with 3–5 missing teeth, and the group with more than 6 missing teeth (P < 0.01) in the fully adjusted model. However there was no statistically significant differences were found between 0 missing teeth and 1–2 missing teeth (P > 0.05). In addition, the results of stratified analysis in the fully adjusted model showed that tooth loss was significantly associated with handgrip strength in participants younger than 60 years of age; however, in participants 60 years of age or older, there was no longer a significant association (Figs 1 and 2).

**Table 2:** Adjusted relationships of tooth loss status with handgrip strength (or handgrip strength per weight).

Number of missing teeth					
	0	1–2	3–5	≥6	*P* for trend^a^
**Males (n = 13983)**	8,400	3,265	1,592	726	-
Handgrip strength(kg)					
Model 1 ^c^	43.9 (43.7, 44.0) ^b^	42.9 (42.7, 43.2)	41.4 (41.1, 41.8) ^f^	38.1 (37.6, 38.6) ^f^	< 0.0001
Model 2 ^d^	43.3 (43.2, 43.5)	43.3 (43.1, 43.6)	42.5 (42.2, 42.8) ^f^	39.9 (39.4, 40.4) ^f^	< 0.0001
Model 3 ^e^	43.6 (42.1, 45.2)	43.6 (42.0, 45.2)	42.7 (41.2, 44.3) ^f^	40.2 (38.7, 41.8) ^f^	< 0.0001
Handgrip strength per body weight(kg/kg)					
Model 1 ^c^	0.56 (0.56, 0.57)	0.56 (0.56, 0.56) ^f^	0.54 (0.54, 0.55) ^f^	0.52 (0.51, 0.53) ^f^	< 0.0001
Model 2 ^d^	0.56 (0.56, 0.56)	0.56 (0.56, 0.56)	0.55 (0.55, 0.56) ^f^	0.52 (0.52, 0.53) ^f^	< 0.0001
Model 3 ^e^	0.57 (0.55, 0.59)	0.57 (0.55, 0.59)	0.56 (0.54, 0.58) ^f^	0.54 (0.52, 0.56) ^f^	< 0.0001
**Female (n = 12292)**	7,866	2,696	1,237	493	-
Handgrip strength(kg)					
Model 1 ^c^	25.7 (25.6, 25.8)	25.5 (25.3, 25.7) ^f^	24.5 (24.2, 24.7) ^f^	23.6 (23.2, 23.9) ^f^	< 0.0001
Model 2 ^d^	25.6 (25.5, 25.7)	25.5 (25.4, 25.7)	24.7 (24.5, 25.0) ^f^	23.9 (23.5, 24.3) ^f^	< 0.0001
Model 3 ^e^	26.1 (24.9, 27.3)	26.0 (24.9, 27.3)	25.3 (24.1, 26.5) ^f^	24.5 (23.4, 25.7) ^f^	< 0.0001
Handgrip strength per body weight(kg/kg)					
Model 1 ^c^	0.44 (0.43, 0.44)	0.42 (0.42, 0.43) ^f^	0.41 (0.40, 0.41) ^f^	0.38 (0.37, 0.39) ^f^	< 0.0001
Model 2 ^d^	0.43 (0.43, 0.43)	0.43 (0.43, 0.43)	0.42 (0.41, 0.42) ^f^	0.41 (0.40, 0.41) ^f^	< 0.0001
Model 3 ^e^	0.43 (0.41, 0.45)	0.43 (0.41, 0.45)	0.42 (0.40, 0.44) ^f^	0.41 (0.39, 0.43) ^f^	< 0.0001

^a^ Analysis of covariance.

^b^ Adjusted least square mean (95% confidence interval) (all such values).

^c^ Crude model.

^d^ Adjusted for age, BMI(body mass index) and WC(waist circumference).

^e^ Adjusted for age, BMI, WC, physical activities, metabolic syndrome, energy intake, dietary patterns, smoking status, drinking status, educational levels, employment status, family income.

^f^ Significantly different from the category of 0 missing teeth (*Bonferroni correction*): *p*<0.01.

## Discussion

### Summary of main findings

This cross-sectional study which was part of a large-scale cohort study revealed that tooth loss was significantly associated with handgrip strength in Chinese adults. From our analysis, it can be seen that numerous factors were associated with tooth loss, including age, employment status, education level, family income, drinking and smoking status, WC, PA, dietary habits, and MS. And, as studies have demonstrated, handgrip strength is closely associated with many factors, such as age and bodyweight (BMI and WC) [6, 45–47]. Thus, we adjusted for age, BMI, WC firstly in model 2, we adjusted for multiple potentially confounding factors sequentially in model 3, the relationships between tooth loss and handgrip strength did not change in both model 2 and model 3. We also compared handgrip strength among different categories of tooth loss and handgrip strength was significantly different between the group with 0 missing teeth, the group with 3–5 missing teeth, and the group with more than 6 missing teeth (*P* < 0.01). We also performed a stratified multivariate analysis. In conclusion, in the fully adjusted model we found: 1) there was a trending association between decreased handgrip strength and fewer retained teeth both in males and females; 2) loss of more than 3 teeth was significantly associated with reduced muscle strength; 3) in the stratified analysis, this association disappeared in elderly participants over 60 years of age.

### Summary of previous findings

In the previous studies which explored whether tooth loss was associated with muscle strength parameters, there was only one study focused on general population [29]. The participants was a German population aged between 30 and 90 years, and the results showed that the number of remaining teeth was significantly associated with handgrip strength when several confounding factors were adjusted. Our findings are in agreement with these results; however, the difference is that we did a stratified analysis, and the association had disappeared in the group over 60 years of age. To the best of our knowledge, most of previous studies focused on old aged adults. Hamalainen et al. compared the handgrip strength by the number of teeth in 193 participants aged 80 years old. This study found the positive association between handgrip strength and the number of remaining teeth in men but failed in women [30]. Two other studies examined the association between tooth loss and handgrip strength in old Japanese population did not find positive results [33, 34]. The results mentioned above are seemingly consistent with our findings in the group over 60 years of age. Previous study has investigated the relationship between tooth loss and the systemic inflammatory reaction and antibody titer of periodontitis bacteria, and the relation between them was an inverted J shape. Moreover, a recent clinical review reported that a steep increase of severe tooth loss (> 9 teeth) around the 70 years of life [48]. In this study, it is may explained why there was no longer a significant association in participants 60 years of age. In addition, dietary pattern analysis was not used in previous studies which examined the effects of overall diet [49].

### Possible biological mechanisms

The mechanisms of the association between tooth loss and handgrip strength is unclear, certain possible explanations may be hypothesized. A well proposed mechanism refers to the inflammatory pathway. As the main reason for tooth loss, oral bacterial infectious diseases are associated with a chronic low-grade inflammation, which can upregulate inflammatory biomarkers including C-reactive protein (CRP), tumor necrosis factor alpha (TNF-alpha), and interleukin-6 (IL-6) [50–52]. There are evidences that handgrip strength are associated with CRP and IL-6 [53, 54], this suggested inflammation was involved in the process of muscle reducing. Firstly, among the relevant signaling pathways, nuclear factor-κB (NFκB) transcription factors plays an important role. NFκB expressed in skeletal muscle was an important mediators of immunity and inflammation which mediating the effects of inflammatory cytokines on muscles [55]. Secondly, periodontitis was associated with insulin resistance and can impair glucose tolerance in general population [56–58]. It subsequently down-regulates IGF-1 though changing in GH/IGF-1 axis bioactivity [59]. IGF-1 is an important anabolic hormone which plays an active role in the maintenance of muscle mass and strength [60]. Thirdly, a close relationship exists between low-grade inflammatory state and the decline of androgens such as testosterone [61]. The secretion of testosterone is controlled by hypothalamic-pituitary (central) and testicular (peripheral), and many experimental studies have shown that IL-6, TNF- alpha and IL-1 beta inhibit the secretion of testosterone by affecting the above components of the gonadal axis [62]. Hormonal dysregulation may concur to the development of disability through decreasing both skeletal muscle mass and strength [63].

Moreover, another possible pathway could be explained, partly by damage of chewing ability brought by tooth loss, which may affect dietary intake and dietary choices, leading to changes in dietary habits [1, 29]. Muscle strength as a nutritional indicator can be affected. Therefore, we adjusted for total energy intake and dietary patterns in the fully adjusted model. Dietary pattern analysis combines nutrients with food consumed, and their combined effects were taking into account, making it the best choice to investigate dietary intake and dietary choices [49]. However, the significant positive correlation between tooth loss and grip strength did not change after the adjustments, which suggested the relationship in this study was independent from dietary patterns and other confounding factors.

## Strengths and limitations

The strengths of the present study include, using a large-scale cohort from a general adult population and not only assessed of missing teeth and handgrip strength but also adjusted for confounding factors as far as possible. Nonetheless, the study also had several limitations. Firstly, we cannot completely exclude residual confounding factors from a cross-sectional study, although we had adjusted for a wide range of demographic characteristics, dietary habits and disease status. There are still potential confounders such as cognitive skills and access to oral health. Secondly, because the examination in the TCLSIH Cohort Study did not include a complete oral health assessment, so we adopted self-reported missing teeth number which may introduce biases that are difficult to control. Moreover, this study only available for the number of missing teeth, without collecting the reasons of when and how each tooth was missing. Therefore, results should be interpreted with caution. Third, we did not obtain the information of hand dexterity needed for oral hygiene may be related to handgrip strength. Therefore, the presented results need to be confirmed by further studies, which including assessment of hand dexterity, dental caries, periodontal diseases and inflammation cytokines such as IL-6 and TNF-alpha. The putative inflammatory pathways linked tooth loss and physical strength warrant further studies to elucidate potential pathogenesis.

Finally, the sample of this study was composed of a relatively young age group, with an average age of 41.6 years. Therefore, our results which only represent this age group and may not generalize to the whole population are needed to validate in other populations in further studies.

## Conclusion

In summary, there was a trend towards less handgrip strength with fewer retained teeth. The underlying causes that could result in both tooth loss and reduced handgrip strength (e.g. inflammation etc.) might lead to the observed association. The stratified analysis between tooth loss and muscle strength in this study may provide proof for previous studies that displayed incongruous results. These results emphasized that controlling the number of missing teeth less than 3 is important for maintaining muscle strength, which can further prevent adverse health outcomes, as well as sarcopenia and frailty in elderly.
